# Wernicke Encephalopathy Associated with Hyperemesis Gravidarum: A Case Report

**DOI:** 10.5811/cpcem.20522

**Published:** 2024-09-29

**Authors:** Beth Kreutzer, Blake Buehrer, Andrew Pelikan, Phillip Rohde

**Affiliations:** University of Missouri, Department of Emergency Medicine, Columbia, Missouri

**Keywords:** Wernicke, hyperemesis, pregnancy, thiamine, case report

## Abstract

**Introduction:**

Wernicke encephalopathy is a clinical diagnosis that requires a high degree of clinical suspicion to recognize. We report a case of a pregnant patient developing Wernicke encephalopathy in the setting of severe hyperemesis gravidarum.

**Case Report:**

The patient was a 22-year-old female 13 weeks pregnant presenting to the emergency department (ED) with neurological deficits after several weeks of hyperemesis gravidarum requiring hospitalization. Exam and workup ultimately revealed the diagnosis of Wernicke encephalopathy. Her symptoms improved after administration of thiamine.

**Conclusion:**

Wernicke encephalopathy is a consequence of thiamine deficiency, commonly seen in patients with alcohol use disorder but also with other causes of nutritional deficiency, such as hyperemesis gravidarum. Wernicke encephalopathy is a clinical diagnosis that requires a high degree of suspicion and is, therefore, often missed in the ED setting. Treatment is supplemental thiamine and management of the root cause for nutritional deficiency.

## INTRODUCTION

The emergency department (ED) manages a large subset of patients who are malnourished to some degree. Critical dietary deficiencies that may be contributing to the presentation pose a diagnostic challenge. Wernicke encephalopathy, caused by thiamine deficiency, is one of those clinical diagnoses that is difficult to diagnose without a high degree of clinical suspicion. Classically, Wernicke encephalopathy is associated with alcohol use disorder; however, any person who is malnourished is at risk for developing this deficiency. Early diagnosis and treatment are imperative to prevent Korsakoff syndrome. Here we discuss a patient who was hospitalized with severe hyperemesis gravidarum and after discharge returned to the ED with neurological symptoms consistent with Wernicke encephalopathy.

## CASE REPORT

A 22-year-old female, gravida 1 para 0 at 13 weeks estimated gestational age, presented to the ED complaining of weakness and confusion. She had fallen in the shower and was unable to ambulate independently. She had blurry vision, difficulty focusing, bulging eyes, headaches, and dizziness. She was intermittently confused and making nonsensical statements. Two weeks prior she had been admitted to the obstetrics service with a urinary tract infection and hyperemesis gravidarum. She was treated with ceftriaxone, intravenous (IV) fluids, and antiemetics and discharged on hospital day five with cefdinir and multiple antiemetics. She had noticed development of these symptoms while still hospitalized.

Prior to the pregnancy, she was healthy without any significant medical concerns. Medications at time of evaluation included cefdinir, doxylamine, ondansetron, pyridoxine, and loratadine. She had no surgical history.

On exam the patient was alert, interactive, and in no acute distress. Vital signs included temperature 36.2° Celsius, respiratory rate 20 breaths per minute, heart rate 109 beats per minute, blood pressure 109/77 millimeters of mercury, and pulse oximetry 100% on room air. She was alert and fully oriented but had repetitive questioning and made frequent nonsensical statements. She had full range of motion in all extremities with normal strength and sensation to upper and lower extremities bilaterally. Her gait was ataxic. She had restricted horizontal conjugate gaze bilaterally and decreased hearing to the left ear. She had no other cranial nerve deficits. Cardiac, pulmonary, and abdominal portions of the exam were unremarkable. She had no rashes, wounds, or lesions on skin exam.

**Image 1:**
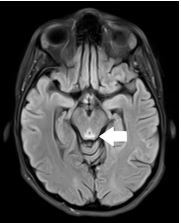
T2 FLAIR Magnetic resonance image demonstrating signal hyperintensity in the periaqueductal gray matter (arrow), consistent with lesion pattern of Wernicke encephalopathy.

**Image 2:**
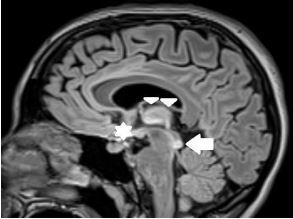
T2 FLAIR Magnetic resonance image demonstrating signal hyperintensity in the periaqueductal gray matter (arrow), medial thalami (triangles), and mammillary bodies (star), consistent with lesion pattern of Wernicke encephalopathy.

On complete blood count the patient was mildly anemic with a hematocrit of 30.1% (reference range 36.0–48.0%), similar as compared to previous. Thyroid stimulating hormone level was 0.014 milliunits per liter (mU/L) (0.4–4.0 mU/L) with free T3 level of 1.28 picograms per milliliter (pg/mL) (1.2–2.7 pg/mL). Complete metabolic panel, magnesium, urinalysis, and drug screens were unremarkable. Computed tomography of the head and neck demonstrated no acute intracranial abnormality. A lumbar puncture was performed with an opening pressure of eight centimeters of water, with no cerebrospinal fluid abnormalities. Magnetic resonance imaging of the brain showed T2 FLAIR signal hyperintensity within the bilateral medial thalami, mammillary bodies, and periaqueductal gray matter, a pattern consistent with Wernicke encephalopathy (Images 1 and 2). Subsequent testing revealed a thiamine level of 29 nanomoles (nmol)/L (70–180 nmol/L).

The patient was admitted to the neurology team for high-dose IV thiamine therapy. She had overall improvement and was discharged on hospital day four with oral thiamine, vitamin D, and prenatal vitamin supplements. She has since given birth to a healthy baby girl and has made a full neurologic recovery.

**CPC-EM Capsule**

What do we already know about this clinical entity?

*Wernicke encephalopathy is a consequence of thiamine deficiency, usually related to alcohol use disorder and as a complication of gastric bypass surgery.*

What makes this presentation of disease reportable?

*Only one previous case report to our knowledge describes the emergency department presentation of Wernicke encephalopathy associated with hyperemesis gravidarum.*

What is the major learning point?

*Diagnosis of Wernicke encephalopathy requires a high degree of clinical suspicion and should be considered in any malnourished patient with new neurological symptoms.*

How might this improve emergency medicine practice?

*Early recognition and treatment initiation by emergency physicians is crucial to clinical improvement and prevention of sequelae such as Korsakoff syndrome.*

## DISCUSSION

Wernicke encephalopathy is an acute manifestation of thiamine (vitamin B1) deficiency. Thiamine is a coenzyme essential to all cells, but it is particularly important for neurons.^1^,^2^ Carl Wernicke first described this encephalopathy in 1881, and in 1940 Campbell and Russell hypothesized thiamine deficiency as the cause.^3^,^4^ Development of brain lesions occurs in regions that have higher demands for thiamine: namely neurons in the thalami, mammillary bodies, tectal plate, and the periaqueductal region.^1^ Some patients who survive their acute encephalopathic episode without treatment go on to develop Korsakoff syndrome, a chronic form of thiamine deficiency that results in severe deficits in memory.^4^,^5^ Other forms of thiamine deficiency include dry and wet beriberi.

Wernicke encephalopathy is classically associated with people who are chronically malnourished secondary to excessive alcohol intake.^2^ However, any condition that leads to malnourishment increases the risk for development of vitamin deficiencies.^4^ Pregnancy is associated with increased demand for thiamine.^6^ There are numerous case reports describing Wernicke encephalopathy in pregnant patients, postulated to be caused by increased metabolic demand from the pregnancy coupled with inadequate intake due to hyperemesis gravidarum.^7^,^8^ However, the literature is sparse regarding diagnosis of Wernicke encephalopathy in the emergency setting, with only one other published case report.^9^

Subclinical thiamine deficiency presents with non-specific symptoms, including headaches, fatigue, abdominal discomfort, and weakness.^4^ Acute deficiency may lead to the classic triad of symptoms: mental status changes, ocular abnormalities (ophthalmoplegia, nystagmus, gaze palsy), and cerebellar abnormalities including ataxia. While only a minority of patients present with the classic triad, our patient presented with all three. Less common symptoms include hypothermia, seizures, hearing loss, and hallucinations.^4^ Late-stage symptoms include hyperthermia, spastic paresis, chorea, coma, and death.^4^

Diagnosis of thiamine deficiency requires a high degree of suspicion, as symptoms may be vague. Wernicke encephalopathy is a clinical diagnosis and should be suspected in any malnourished patient presenting with suggestive neurological symptoms. The Caine criteria have been proposed to help predict the likelihood of Wernicke encephalopathy, requiring at least two of the four following findings to support the diagnosis: dietary deficiencies, ocular signs, cerebellar dysfunction, and altered mentation or memory impairment.^10^,^11^ While serum thiamine levels may be measured, these measurements may not be reliable.^12^,^13^ Magnetic resonance is the imaging of choice for diagnosis of Wernicke encephalopathy, as it shows distinct patterns of alterations in the typical regions affected by the deficiency.^14^,^15^

Treatment for Wernicke encephalopathy is thiamine repletion. Standard prenatal vitamins have 1–2 milligrams thiamine, which would be insufficient in the pregnant patient who develops Wernicke encephalopathy. Therefore, pregnant patients who are struggling with hyperemesis should be treated with additional thiamine supplementation.

## CONCLUSION

Wernicke encephalopathy is a consequence of acute thiamine deficiency, often associated with alcohol use disorder but can occur with any cause of nutritional deficiency. There are several case reports describing patients with hyperemesis gravidarum who developed Wernicke encephalopathy, but only one report regarding ED presentation and diagnosis. Wernicke encephalopathy is a clinical diagnosis that requires a high degree of suspicion; therefore, it should be suspected in any patient who is malnourished and displaying neurologic symptoms.
